# Lectures on Scarlet Fever

**Published:** 1851-10

**Authors:** Caspar Morris

**Affiliations:** Late Lecturer on Practical Medicine in the Philadelphia Medical Institute


### MEDICAL EXAMINER,

AND

### RECORD OF MEDICAL SCIENCE.

NEW SERIES.—NO. LXXXII.—OCTOBER, 1851.

### ORIGINAL COMMUNICATIONS.

*Lectures on Scarlet Fever.* By CASPAR MORRIS, M. D., late Lecturer on Practical Medicine in the Philadelphia Medical Institute.

### LECTURE VIII.

While, as we have seen, death occasionally defies our best directed efforts, and terminates the course of even *simple* scarlet fever, either as the result of some unexpected local inflammation, or, more often, of one of the sequelæ of the disease; and with greater, though still varying frequency, occurs in all stages of the *anginose* variety—the *malignant* form more often sets at defiance the utmost energy of treatment, even when directed by the most consummate skill. Your memory will not have failed to retain the melancholy picture of its ravages in the earlier period of the settlement of this country, as drawn by Dr. Kearsley; and similar vestiges of its progress have been left in all parts of Europe, as well as in every age, of the diseases of which we possess any history sufficiently accurate to enable us to distinguish them clearly. The very term *malignant*, conveys, even to the unprofessional ear, the idea of great danger, and in the language of medicine is meant to express a great tendency to the prostration of the vital forces. The knowledge of the lethal character of this form of the disease, should, however, lead to no despondency on your part, but rather stimulate you to more determined effort to rescue the patient, and increase the triumph of medical science. There are many other diseases which partake more or less, at different times, of this tendency to prostration. Between them and malignant scarlet fever, there is this, however, very important point of difference, that in this the local lesions are very severe, and often in themselves sufficient to cause death, even though the vital energy were not impaired by the peculiar character of the case.

A theory which taught that in this, as in the kindred forms of fever characterized by the same tendency to rapid decline of vital power, there was a previous stage of excitement of greater or less intensity, upon which the subsequent depression depended, not as a mere sequence, but as the effect upon a cause, was at one time generally received, and exerted a most deleterious influence on the interests of medical science. Discarded now by most authorities from its position in connection with other fevers, it still exhibits some lingering traces of its influence in a few of even the more modern writers on this disease. Those who adopted this view, recommended in strong terms the prompt resort to an antiphlogistic treatment of the early stage, under the impression of the fallacious hope that by such measures they would diminish the primary reaction, and thus avert the consequent depression; and there are not wanting authors of even a recent date, who still teach this erroneous view; and who strongly recommend the prompt resort to bleeding, vomiting, and purging. When called early to these cases, they do not hesitate to direct an antimonial emetic, followed by an active mercurial purgative, nor do they shrink from a prompt and decided bleeding if these fail. They assert that "this treatment, if adopted at the onset of the symptoms, will generally, not only moderate the fever, but shorten the duration and violence of the disease."

Nor is this view, and the practice founded on it, confined to European physicians. One of the most authoritative of our own writers on the diseases of children, my friend Dr. Condie, has adopted them to their full extent, and claims for his practice a degree of success which justifies the positive terms in which he commends his views. "In a table of two hundred and sixty-eight cases, occurring during different epidemics of considerable extent and severity, and treated on the plan laid down by him, (into which bleeding, calomel in full doses, and castor oil with spirits of turpentine, as purgatives, enter,) two hundred and twenty-three recoveries, and forty-five deaths are enumerated." Dr. C. asserts of the treatment he recommends, that "when judiciously carried into execution, it is calculated to disarm the disease of its 'malignancy' and to prevent the necessity for a resort to powerful cordials, tonics and antiseptics, in the advanced period of the attack, to remove the putrid symptoms when they show themselves." "The bold and indiscriminate use of the lancet," Dr. C. "strongly objects to; but of the good effects a cautious use of blood-letting in the manner and under the circumstances directed, is calculated to produce, he speaks from actual observation." "It is," adds Dr. C. "unquestionably, in a large number of cases, the only 'restorative and tonic' upon which any confidence can be placed." In thus setting before you the testimony of Dr. Condie, I have designed to furnish you with the strongest statements in favor of a well directed antiphlogistic treatment.

I need not repeat what I have already told you when treating of the other forms of this disease, nor inform you again that, believing the primary influence of the cause to be depression, I cannot adopt the view which assigns to its first impression a sthenic character. If bleeding, and antimonials, and purgatives are inappropriate to the simple anginose form, much less are they required in that now under consideration, and Dr. R. Williams, after a careful investigation of the history of the epidemics which have prevailed from 1763 to 1834, asserts, with regard to scarlet fever in general, "that the chances of recovery are diminished by the practice of bleeding, in the ratio of nearly four to one, as compared with the chances supposing the patient not to have been bled."

With this expression, my own observation is coincident, and you will find my views sustained by not a few authorities of deserved repute. Dr. Fothergill, than whom a more accurate, careful observer, or more honest writer, cannot be found, when he sums up his views of the practice and treatment of this disease, of which it is evident he saw the *malignant form*, asserts, "that a cordial, alexipharmic, warm regimen has been found by experience to be of the most use in such cases," and condemns as injurious, "bleeding, purging, and antiphlogistics liberally employed." He says: "In some of the first cases I met with, the quickness of the pulse, the degree of heat, the apparent inflammatory redness of the eyes and face, and pain in the head, sometimes urged me to order bleeding, especially if there were any marks of plethora; but in these cases it did not appear to have any advantageous effects; so that notwithstanding the vehemency of the symptoms above mentioned, it seems proper in general to omit this evacuation."

Dr. Kearsley, speaking of the disease as it appeared in Philadelphia in 1746, says: "The pulse was generally full and quick, yet attended with some remissions and even sinkings, but it most commonly kept up those deceiving strokes which sometimes but very improperly indicate the use of the lancet." . . . . . "Although most of the symptoms in the beginning of this disease, as well as the fulness of the pulse, appeared to point out to us the necessity of bleeding, yet, should we have complied with the indication, it would have proved a fatal error." That this opinion was the result of observation is proved by the remark, that "the blood which has been drawn away in these cases has been often observed to have a tenacious glue upon the surface, and yet, nevertheless, it has been found underneath to be broken, loose and divided in its texture, and has also thereon very evident marks of a putrid gore which must and does increase by bleeding." "One bloodletting may make a difference of a change from a texture that, with proper management, would have carried a patient through the sickness with safety, to no texture at all, but a total dissolution of all animal fluids which must in course terminate in death. Of such vast consequence is this one article of bleeding, that it has been my choice to give this caution against it in the strongest terms." Dr. Burrows in an interesting article on this disease, in the library of Practical Medicine, remarks of the malignant form, that, "it quickly indicates its formidable nature by the sudden depression of the vital powers. If bloodletting from the arm be a remedy of doubtful propriety in the two former varieties, it is here hazardous in the extreme." Dr. Chapman says, "The abstraction of blood appears to be required by the loaded state of the organs, and contraindicated by the depression of the vital energies. My own conviction is, that it should not be hazarded, unless reaction is pretty firmly established, the circulation in some force, and the skin warm, and even then is to be resorted to with extreme circumspection." And with regard to the general applicability of bloodletting as a remedy in any form of Scarlet Fever he says: "it is true that the loss of blood has no direct curative tendency in the disease, it only abating action, without changing or subverting it; and is usually not well borne to any extent. It is not safe to detract it with the same freedom as in more purely phlegmasial affections, or perhaps, to the amount that the existing indications in the case would seem to demand. Collapse, frightful and sometimes even fatal, I have repeatedly seen to result from an abuse of the practice, and it is always hazardous in an advanced stage of the disease."

Dr. J. Forsyth Meigs, in his able work on the diseases of children, after producing the testimony of many distinguished writers on this disease to the doubtful tendency of a resort to bloodletting, says: "On the whole, it is clear, I think, that the weight of evidence is against bloodletting to any considerable extent, in grave cases. If used at all, it is only to be used in the earliest period, and even then with great caution. My own opinion, derived from personal experience, is as follows:—I believe that I have seen general depletion useful in several cases of the regular form, in which there was a tendency to the grave form, shown by the presence of excessive reaction, and still more by great jactitation and irritability, alternating with drowsiness and delirium. But in those sudden attacks of the disease, in which it assumes from the very start the terrible symptoms which threaten extreme danger to the patient, in which we find the child within a few hours of the onset, delirious or comatose, or laboring under convulsions, convulsive movements or contractions, in which the eruption is imperfect or scanty, or copious and of a deep livid tint; in which in other words there are either strongly marked ataxic or adynamic symptoms, general bloodletting has never seemed to me at all advantageous, and I have several times feared that it had been injurious. As to leeches, I have never known them to be really useful but in one case, and in that they were used sparingly, and after an interval of two days. In all other cases they appeared to be without any effect."

I might swell largely the list of authors who have either condemned depletion entirely, or expressed their doubt of its fitness to the treatment and proposed its employment with caution; but I have adduced testimony enough to prove to you that observation, places depletion among the remedies of doubtful value, to say the least of it, and thus confirms the view, derived from reasoning on the nature of the disease, its origin and course, which I have endeavored to impress upon you.

But though blood-letting, antimonial emetics, and antiphlogistic treatment, are all, for the same reason, excluded from our list of remedies in the malignant as well as the other forms of scarlet fever, a simple emetic of ipecacuanha, or infusion of Eupatorium perfoliatum, the thoroughwort or bone-set, of our own meadows, may be given in the commencement of a case, with decided advantage. It evacuates the primæ viæ, and produces a strong determination toward the surface, without leaving any secondary depression of the vital forces, or exhaustion, such as follows the use of antimonials.

The action of this having been accomplished, your attention should at once be given to the support of the vital power, which will be found flagging from the very commencement in many cases, and to the arrest of the local lesions in the fauces and pharynx. The capsicum is here an agent of great value, acting at once to the fulfilment of both indications. We are indebted to the West India practitioners for the introduction of this remedy to our notice in this disease. We have been taught to look upon it as a simple stimulant, without any direct impression on the general system, and that it acts upon this in a secondary manner, through the increased energy it communicates to the stomach. My observation of its effects in these cases of scarlet fever, does not permit me to adopt this as the only mode in which it promotes the recovery of the patient. It must stimulate the exhausted nervous forces, if no farther than to enable the stomach to appropriate properly the nourishment presented to it. While it thus acts advantageously on the general system, the local impression on the throat is equally beneficial. Though the first sensation produced is one of increased heat, this soon passes away, and is followed by a sense of relief from the tension and soreness which aggravate, so materially, the sufferings of the patient.

I have conducted many cases to a favorable conclusion by these remedies alone, giving the capsicum in the manner suggested, when treating of the form of the disease last under notice, and supporting the strength of the patient by animal broth.

Purgatives have been recommended in this as in the other forms of the disease, even by those who condemn other antiphlogistic treatment; especially calomel, either in combination with those articles usually united with it to secure its prompt action, or followed by castor oil and spirits of turpentine. The object proposed by this treatment, is to promote a free secretion from the viscera, and, as is supposed, remove a load which is thought to oppress the vital energy. The reasoning is, I think erroneous, and the practice founded on it pernicious. The congested condition of the vascular system, and the suspension of secretory action, are dependent on the diminution of the vital forces, and not the cause of that depression; and it were as contrary to common reason to bleed a porter struggling to support a load beyond his power, as to resort to any remedy which diminishes vital force in the malignant form of scarlet fever. In no instance have I seen any advantage result from the use of a mercurial in any form or stage of the disease. Mr. Colden and Dr. Kearsley, however, coincide in the assertion that Dr. Douglass of Boston commends its use, in strong terms, in the malignant form, and even urged it to the extent of salivation. I have not been able to procure a copy of the original work of Dr. Douglass. He wrote, however, at a period when the influence of mercury was thought to be almost omnipotent, and when it was employed with but little discrimination or knowledge of its character, in almost all forms of disease.

My mode of treating these cases, may be illustrated by the record of my experience in one family. It was that of a widow who had been compelled to leave her children without protection, while she returned to England to secure a bequest. The oldest was a girl of about fifteen years.—She and two others were seized simultaneously with scarlet fever. I found her lying, insensible, on the bed, the whole surface of a mulberry hue, the pulse too frequent to be counted, perfect stupor and extreme jactitation, the throat swollen so as entirely to prevent deglutition, the fauces covered with ash colored deposit, a fœtid ichor distilling from the nostrils, the eyes injected and upturned. The two other cases presented the usual marks of the severe anginose form. I at once secured the services of a judicious nurse, and in order to call out the utmost exertion of her care and effort, I told her, that though severe, the worst case was not hopeless, and that I was so conscious that the best medical advice would be useless without the aid of good nursing, that I was willing she should have all the credit of her recovery if it were accomplished. I furnished her with a syringe, and introduced a stomach tube through the nostrils. Wine whey, beef broth, highly seasoned with capsicum, and quinine, were injected into the stomach at stated intervals, while the solution of sulphate of copper, and compound capsicum infusion were employed as local applications to the throat, by means of a syringe. Convulsions occurred within a few hours; three days she lay in the condition described, the only evidence of consciousness afforded, was in the resistance she opposed to treatment. On the fourth day the jactitation diminished, and there was a manifest abatement of the stupor, and much to my gratification, she recovered without any sequelæ being developed. The other cases were treated in the simple manner described when speaking of the anginose form, and with an equally happy result.

The application of cold to the surface, and the use of ice internally, has been advocated by some authors even in this form of the disease. I need hardly say that the sedative impression of cold, is as much to be avoided as that of any other agent, but where the skin is hot and dry and the eruption is very abundant, great benefit results from the tepid sponging, and the addition of a small quantity of vinegar or whiskey to the water is advantageous. In the first few hours of a case, or when there is still sufficient intelligence remaining to enable the patient to manage the ice, it may be employed for the mitigation of the suffering from the angina. Several authors speak of fatal collapse following the injudicious resort to external cold.

Dr. Meigs furnishes us with a quotation from Dr. Currie, to whom we owe the introduction of this treatment, which proves that he did not consider it appropriate. The cases to which he refers as "purpurata," are those which I include under the head of malignant, and to such, Dr. C. says, "the cold effusion is scarcely applicable, and tepid effusion makes little impression. In my experience, indeed, all remedies have been equally unsuccessful. It outstrips in rapidity, and it equals in fatality the purple confluent small-pox, to which it may be compared." The internal use of ice in these cases is too often forbidden by the stupor and delirium. Where these do not prevent, it may be resorted to with the same advantage as in the common anginose form. Where there is decided ulceration and sloughing of the throat, great benefit may be derived from the injection of chloride of soda. I have never used it myself, having always relied upon the same injections in these cases as in the more severe instances of the anginose variety. The capsicum wash there mentioned, is best suited to this form of the disease. The internal use of a weak solution of the chloride of soda has been strongly recommended by Dr. Jackson of Northumberland, who reports many cases in his own practice, and that of his friends, which recovered under the use of this remedy and ice. Its antiseptic qualities have caused it to be employed internally in this as well as in other forms of adynamic fever, but, I believe, with little benefit which could be fairly attributed to this remedy alone.

No attempt should be made to separate the sloughs or detach the deposits from the mucous membrane of the throat, either by mechanical means or by harshly stimulating washes. Fatal hemorrhage is sometimes brought about by this rude interference, or the tendency to gangrene aggravated by it.

The local abstraction of blood from the neck in these cases, can not be too strongly condemned. It can no more arrest the progress of the inflammation of the fauces, than it can that of an external part tending to gangrene. The bites of the leeches do not heal, but continue to permit the leakage of a thin bloody serum, till they take on a low grade of inflammation themselves, which results either in sphacelus, or spreading ulcers. The remark is equally applicable to the use of blisters, which almost always, either slough or ulcerate, and have not unfrequently proved the cause of death even after the sufferer had struggled through the disease for the relief of which they were employed.

Carbonate of ammonia has been highly commended by some authors in this form of disease. One insuperable obstacle to its use, is found in the difficulty of deglutition. Even in those forms of low fever which are free from angina, the pungent character of the remedy makes it difficult of administration, and where there is much swelling or ulceration, it would be impossible that it could be swallowed, especially by a child.

Musk, too, and camphor, have their advocates in those cases where the nervous symptoms predominate. I do not believe they possess any special virtues which entitle them to favor, and prefer depending on a few tried and proved friends, to the resort to many other articles of questionable powers. The rational indications are met by those I have recommended, and on them you may safely rely.

In those *irregular* cases which I have described, it is impossible to direct any plan of treatment. There is no specific for scarlet fever, even in its well developed forms. You will observe, I have endeavored to indicate to you the symptoms, and to deduce from them a treatment based on the known rules which regulate the human economy in health and disease. Where these laws cease to exert a control, the physician must be guided by his own discretion in each case as it presents itself.

Warm, stimulating, spirituous baths, or vapor baths, may be employed to invite the diseased action to the skin, and thus to liberate the central nervous system from the load by which it is oppressed; and where there is much tendency to collapse, warm wine whey or brandy may be given internally.

In those cases where the skin is hot and dry and yet devoid of eruption, and there is great restlessness, I have used the Dover's powder with advantage. Capsicum, too, in some cases of this kind, has appeared to confer benefits which might be supposed almost to entitle it to the character of a specific; and should never be omitted.

It may not be amiss to furnish you with a succinct statement of the plan of treatment, which with the reasons on which it is founded, I have thus spread before you.

*Simple* Scarlet Fever without complication, may be confided to cool drinks, cool air and sponging the surface, with light diet. Where the fever is more intense, a weak solution of carbonate of soda, with a small quantity of sweet spirit of nitre, may be given.

During convalesence, and until the skin has resumed its healthy action, the most vigilant care is required to avoid too great license in diet, and exposure to atmospheric changes.

When the case is complicated by the occurrence of delirium, convulsions, or irregular muscular contractioms, these symptons may best be combated by cold to the head, and sponging the surface.

The *anginose* affection is best treated by the free use of ice, and gargles of cold water, internally; and emollient poultices or wet cloths externally; the general treatment being the same as in the simple form, and the caution about diet and exposure equally, or even more important.

The *malignant* form requires that the first symptoms of failure of vital powers should be met by a prompt resort to the use of capsicum, quinine, wine, and nourishment; while the local affection is to be treated in the same manner, and by the same remedies as in the anginose form. In the very beginning ice may be used; but when the ash colored deposits appear, or sloughing commences, nothing will equal the benefit you will derive from the use of the capsicum infusion, alternated with a solution of sulphate of copper; care being taken in using the latter, to avoid passing so large an amount of it into the stomach, as shall produce vomiting, and thus depress fatally the already exhausted forces.

Free ventilation is of the utmost importance in all the forms of this disease; but in the malignant form its value is beyond estimation. Nor is there any danger from the influence even of cool draughts, so much dreaded by parents and nurses during the progress of the primary fever.

I am fully aware that many condemn the views I have here presented to you, and that it is not uncommon to find the advocates of the antiphlogistic treatment, attributing the mortality of this disease to the stimulating treatment I have thus recommended. Improperly employed it is certainly pernicious. It should never be resorted to except where the feeble pulse, languid circulation, livid or violet color of the eruption, and sloughing throat give unmistakeable evidence of the failure of vital power. The resort to the treatment appropriate to this state from an apprehension of its approach, is, indeed, likely to induce it. It is even more to be deprecated than the opposite extreme. Where bleeding, the application of cold, or the exhausting treatment has reduced the patient, there is still room for hope that reaction may be produced by the resort to the proper remedies. But where the nervous energy has been exhausted by the addition of artificial stimulation to the febrile excitement, a state of prostration from over excitement is induced for which we have no remedy. While, therefore, I desire to caution you against the resort to those measures which directly exhaust the vital power, I would enter an equally strong protest against the premature employment of stimulating remedies. If, as in the case I have described, the malignant symptoms are present from the first, adopt immediately the stimulation necessary to counteract their tendency ; but do not let the fear of its future occurrence induce the premature resort to them as a preventive. This, like bleeding beforehand to avoid inflammation, will only precipitate or make more certain the result which is dreaded. These remarks are especially applicable to the use of quinine. The wine whey is transient in its influence, the capsicum gives rise to no febrile reaction, but the quinine produces a more permanent impression upon the nervous system, and has a stronger tendency to excite local inflammation. Properly applied, it is the sheet anchor of our hope ; the very power which renders it so, causes it when misused to be most pernicious. It should be given in full doses at long intervals, leaving the stomach free to receive the animal broth, wine whey, or brandy. These latter are most conveniently given in arrow root or some of the usual farinaceous articles of diet.

Before passing to the consideration of the sequelæ of the disease, and the proper treatment of them, some notice must be bestowed on the question, how far it is possible to remove the susceptibility to the impression of scarlet fever. It is now many years since it was announced that the administration of small doses of belladonna to those who were exposed to the influence of this cause, whether epidemic or contagious, produced an almost entire immunity. The source from which the suggestion came, though entirely destitute of authority, was less calculated to excite suspicion than the reasoning by which the assertion of its power was supported.

Its use was first suggested by Hahneman, who supposed that in the affection of the throat, and redness of the face, produced by an *excessive dose* of this narcotic poison, he discovered a train of symptoms similar to those of scarlet fever, and hence inferred that its use in *infinitesimal doses* would produce a condition similar to that caused by the miasm, and prevent the real disease. No reasoning could be less logical. Knowing how many escape when exposed to the influence of this specific poison, much incredulity as to the effect of the remedy has been entertained. Still it has been extensively tried, as well in this country as in Europe, and the testimony to its prophylactic influence cannot be disregarded.

MM. Rilliet and Barthez, gave us the report of Gumpert, who employed it in more than twenty families with entire success. Berndt found that of one hundred and twenty-four children to whom it was administered during exposure, only fourteen took the disease; and Hillenkamp had still better results, only five having sickened out of one hundred and twenty who took the belladonna. Our own medical journals contain reports of the same favorable character. Dr. Condie, however, asserts that having tried it in repeated instances, he never found it to produce the slightest effect in mitigating the character or preventing the occurrence of scarlet fever.

There are so many sources of doubt, that it is impossible, in the present stage of our experience, to give a positive opinion. The only field for a crucial experiment is some large institution for orphans or destitute children, where it can be tried, while all other means of prevention are avoided. Larger numbers than can be found in private families, and more careful observation are necessary to settle this point. In the mean time, though the reasoning of Hahneman is fallacious, it is quite possible that the influence on the nerves of organic life, of a slight narcotic, may so preoccupy them that they shall not yield to the impression of the epidemic or contagious principle. If it be proven to have this effect in scarlet fever, it should be equally available in the case of other diseases which are diffused by the same agency. Professor Chapman long since drew attention to the effect of the occupation of the stomach by food, in preserving those who were exposed to miasmatic impressions. It were impossible to keep the stomach always filled with food ; but the nervous system may be kept constantly affected by the narcotic, and, if given in quantities sufficiently minute, without inconvenience. I have never tested the effect myself, not having had an opportunity which was sufficiently guarded from sources of uncertainty. Though I have thus drawn your attention to it on account of the positive assertions of its advocates, and have endeavored to exhibit the manner in which, if at all useful, it operates, I have no faith in the prophylactic power of this, or any other agent. The mode of exhibition recommended, is to suspend *three grains* of the extract of belladonna in *one ounce* of water; of this, *two* or *three drops* are to be given twice a day, to a child under twelve months old, adding one drop for each additional year of age.

(To be continued.)

